# Relative potency in SPT of solution and tablet SLIT allergen extracts of Timothy grass pollen from 2 European manufacturers compared to a US reference extract

**DOI:** 10.1186/1939-4551-8-S1-A287

**Published:** 2015-04-08

**Authors:** Désirée Larenas-Linnemann, Robert Esch, Jaswinder Singh, Juan José Matta, Nelson Rosario Filho, Jorge Maspero, Alexandra Michels, Ralph Mösges

**Affiliations:** 1 Hospital Médica Sur, Mexico; 2 Greer Inc. Lenoir, USA; 3 Imsie Center of Investigation and Statistics, Germany; 4 Hospital Siglo XXI, Brazil; 5 University of Parana, Brazil; 6 Fundación Cidea, Brazil; 7 University at Cologne, Germany

## Rationale

To compare the relative potency in skin tests of solution and tablet extracts of Timothy grass pollen (TIM) of 2 European manufacturers (ALK-Abelló, Stallergènes), with an FDA approved extract (REF) of 10,000BAU/mL.

## Methods

This is a prospective, multicenter, triple blinded, randomized study in which the *in vivo* extract potency was determined, based on the wheal size obtained in TIM allergic patients. The four tested TIM extracts were: Soluprick, Staloral 300IR, and Grazax and Oralair 300IR dissolved in 1mL 50% glycerin (under GMP standards). The SPTs were carried out in quadruplicate with the concentrate extracts and three serial half-log dilutions, and +/- controls. The study took place at study sites with different climatologic conditions. To determine if there exists a statistically significant difference between the relative potency of the TIM extracts a parallel line bioassay was carried out using the mean surface of the four wheals of the SPTs per extract and per concentration (Wilcoxon, Asymp. Sig.(2-tailed)). Based on the wheal sizes of the concentrate extracts in relation to the REF, BAU values were calculated.

## Results

Differences in wheal size between concentrate extracts reached statistical significance for all, except Soluprick-REF. The calculated BAU compared to the REF values for both solutions were between 11,300-16,300BAU/mL and the tablets varied between 4200-7300 BAU.

## Conclusions

Based on SPT whealsizes grass-tablets seem to have a higher relative potency than the previously published value of 2800BAU (which was based on in vitro testing). There is a statistically significant difference (p=0.011) between the allergen concentration as measured in SPT of both tablets. Probably other factors apart from the precise allergen content of the tablets and their potency determine the efficacy of sublingual immunotherapy.

